# Targeted Delivery of CNS‐Specific Hesperidin as a Leptin Sensitizer for Treating Obesity‐Associated Sleep‐Disordered Breathing

**DOI:** 10.1002/advs.202506182

**Published:** 2025-09-09

**Authors:** Yixuan Wang, Qingyu Zhao, Qingfeng Zhang, Xiaofeng Wu, Xu Liu, Shujuan Wu, Hailing Liu, Beini Zhou, Shiqian Zha, Dong Zhao, Gang Deng, Ke Hu

**Affiliations:** ^1^ Department of Respiratory and Critical Care Medicine Renmin Hospital of Wuhan University Wuhan Hubei 430060 China; ^2^ Department of Neurosurgery Renmin Hospital of Wuhan University Wuhan Hubei 430060 China; ^3^ Department of Pulmonary and Critical Care Medicine Taihe Hospital Hubei University of Medicine Shiyan Hubei 442000 China

**Keywords:** endoplasmic reticulum stress, hesperidin, leptin sensitizer, nanoparticles, sleep‐disordered breathing

## Abstract

Obesity‐associated obstructive sleep apnea (OSA) highlights the need for effective therapies. Hypothalamic endoplasmic reticulum (ER) stress contributes to leptin resistance in obesity. Although hesperidin (HE) modulates ER stress and oxidative pathways, its low bioavailability limits clinical use, its role in OSA is unknown. Self‐assembled HE nanoparticles (HE NPs) are developed to address this. HE NPs are synthesized via solvent emulsification‐evaporation and tested in diet‐induced obese (DIO), lean, and ob/ob mice. In DIO mice, HE NPs reduce food intake, body weight, and plasma leptin while mitigating hypothalamic ER stress and boosting STAT3 phosphorylation, without toxicity, and they outperform HE and PLGA NPs. In ob/ob mice, HE NPs lower PERK phosphorylation and, with leptin, further suppress weight gain and food intake while enhancing STAT3 signaling. Combining HE NPs with leptin in DIO mice amplifies weight loss versus HE NPs alone. HE NPs also improve sleep‐disordered breathing, lowering the apnea index during non‐rapid eye movement sleep (17.1±3.5 to 9.3±3.0 events h^−1^, *P* < 0.01) and rapid eye movement sleep (22.4±5.6 to 12.8±3.9 events h^−1^, *P* < 0.05). The results demonstrate that HE NPs improve metabolic dysfunction and OSA in obesity by reducing ER stress and restoring leptin sensitivity, offering a novel therapeutic strategy.

## Graphical abstract

Hesperidin nanoparticles (HE NPs) effectively combat obesity and sleep‐disordered breathing in mice by enhancing leptin sensitivity. Through suppression of hypothalamic endoplasmic reticulum stress and restoration of leptin signaling, HE NPs promote weight loss and improve breathing during sleep. These findings highlight HE NPs as a promising therapeutic approach for obesity‐related metabolic and respiratory complications.

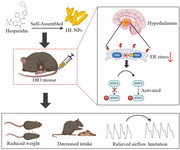

## 1 Introduction

Obstructive sleep apnea (OSA) is a prevalent respiratory disorder characterized by recurrent airway collapse during sleep.^[^ ^1^ ^]^ Its prevalence continues to rise due to the obesity epidemic and population aging.^[^ ^2^ ^]^ OSA not only leads to oxygen desaturation and sleep fragmentation but also increases the risk of cardiovascular and metabolic diseases, contributing to neuropsychiatric complications, significantly impairing patients' quality of life, and imposing substantial socioeconomic burdens.^[^ ^3^ ^]^ Obesity, as a major risk factor for OSA, promotes disease development through multiple mechanisms: fat deposition alters upper airway anatomy, increasing collapse risk;^[^ ^4^, ^5^ ^]^ abdominal fat reduces lung volume, diminishing pharyngeal longitudinal traction;^[^ ^6^ ^]^ and leptin resistance impairs ventilatory regulation, exacerbating apneic events.^[^ ^7^ ^]^ However, current treatment strategies face significant challenges: lifestyle interventions struggle to maintain long‐term weight loss (success rate <10%),^[^ ^8^ ^]^ and the adherence rate to continuous positive airway pressure, the first‐line treatment, is only 30%‐60%.^[^ ^9^ ^]^ These limitations underscore the urgent need for novel therapeutic strategies.

Leptin, a key hormone secreted by adipose tissue, crosses the blood‐brain barrier to act on the hypothalamus and medulla,^[^ ^10^ ^]^ exerting appetite suppression and energy expenditure enhancement through the long‐form leptin receptor and downstream STAT3 signaling pathway.^[^ ^11^ ^]^ It also plays a critical role in respiratory regulation: studies show that leptin‐deficient ob/ob mice exhibit reduced hypercapnic ventilatory response sensitivity,^[^ ^12^ ^]^ which can be corrected by leptin supplementation, improving upper airway reflexes and reducing inspiratory flow limitation during sleep.^[^ ^13^ ^]^ In diet‐induced obesity (DIO) mice model, leptin treatment also reduces oxygen desaturation events during REM sleep and increases ventilation in both non‐rapid eye movement sleep (NREM) and rapid eye movement sleep (REM).^[^ ^14^ ^]^ However, the widespread phenomenon of leptin resistance in obese populations severely limits leptin's therapeutic potential, with mechanisms closely linked to endoplasmic reticulum (ER) stress in hypothalamic neurons: sustained ER stress disrupts the unfolded protein response, interfering with leptin signaling and reducing hypothalamic leptin sensitivity.^[^ ^15^ ^]^ These findings not only establish leptin as a potential therapeutic target for obesity‐associated OSA but also suggest that targeting ER stress to improve leptin resistance may represent a novel therapeutic strategy.

Hesperidin (HE), a flavanone glycoside abundantly found in citrus fruits, exhibits diverse pharmacological activities. Studies demonstrate that HE not only possesses antioxidant, anti‐inflammatory, and immunomodulatory properties but also effectively alleviates ER stress and inflammatory responses by regulating multiple signaling pathways, including MAPK, NF‐κB, and TNF‐α.^[^ ^16^, ^17^ ^]^ Specifically, HE mitigates sodium fluoride‐induced testicular toxicity by reducing ER stress^[^ ^18^ ^]^ and protects against paclitaxel‐induced testicular tissue damage, significantly alleviating inflammation, apoptosis, and ER stress levels.^[^ ^19^ ^]^ Despite its potent ER stress‐modulating capabilities, HE's application in OSA treatment remains unexplored. Moreover, HE faces significant clinical challenges: poor water solubility (5.92 ± 0.49 µg mL^−1^ at 25 °C), low partition coefficient, and poor cell membrane permeability, ultimately resulting in suboptimal bioavailability.^[^ ^20^, ^21^ ^]^ To address these issues, nanotechnology offers promising solutions. Our team's previous research has demonstrated that self‐assembled nanoparticles (NPs) of natural compounds can significantly enhance drug bioavailability.^[^ ^22^ ^]^

Building on this background, this study innovatively developed a hesperidin nanoparticle (HE NPs) delivery system. By leveraging small‐molecule self‐assembly technology, HE NPs not only overcome the limitations of HE's low bioavailability but also fully exploit its ER stress‐modulating effects. We systematically evaluated the regulatory effects of HE NPs on leptin resistance and thoroughly investigated their role in improving metabolic and respiratory functions in a sleep‐disordered breathing (SDB) mouse model, providing a novel therapeutic strategy for OSA treatment.

## 2 Results

### 2.1 Synthesis and Characterization of HE NPs

**Figure 1:**
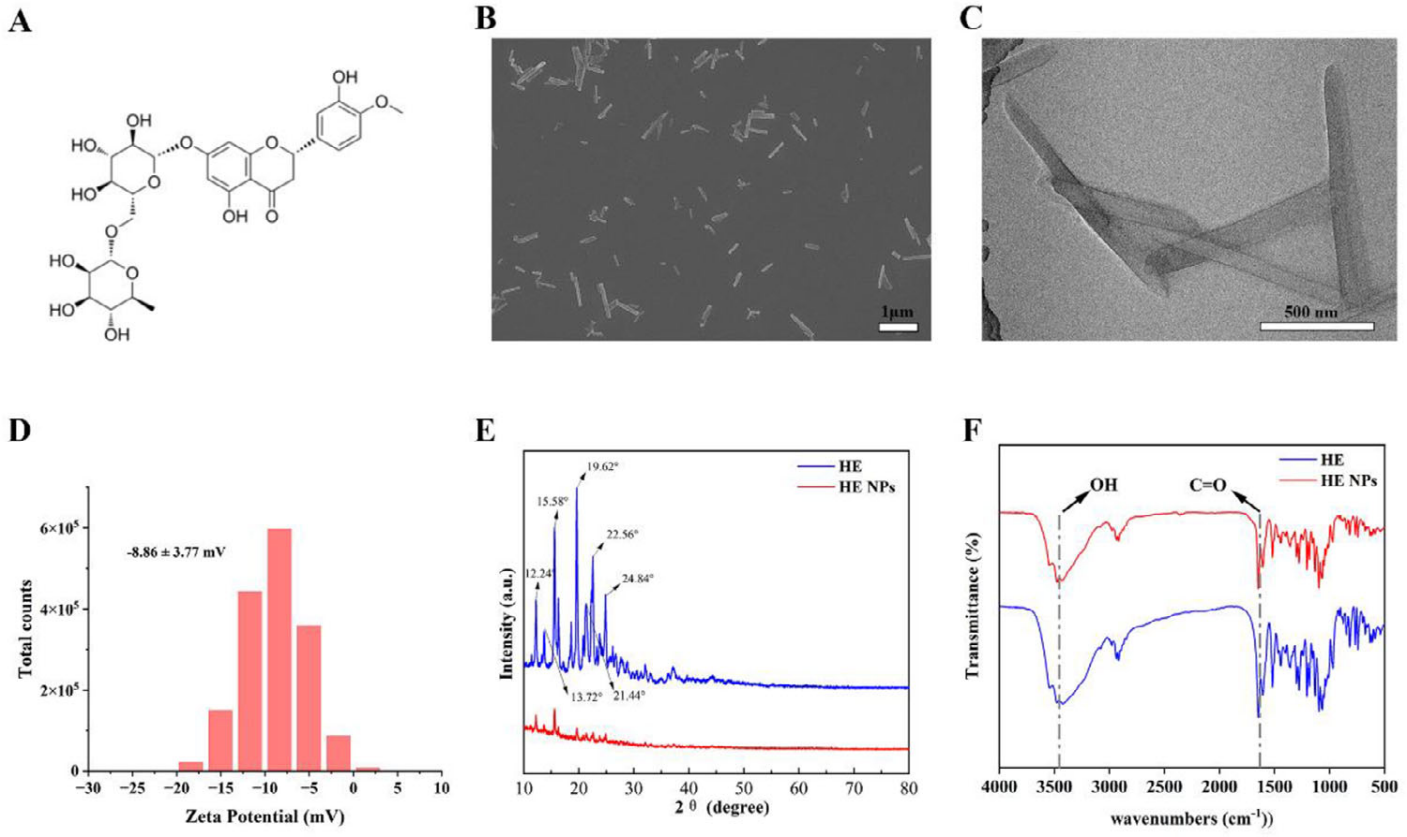
Construction and characterization of HE NPs. A) Molecular structure of HE NPs. B) Representative SEM image showing the morphology and size of freeze‐dried HE NPs (scale bar = 1 µm). C) Representative TEM image illustrating the morphology and size of HE NPs (scale bar = 500 nm). D) Zeta potential of HE NPs determined by Electrophoretic light scattering (ELS). E) Comparison of X‐ray diffraction (XRD) patterns between free HE and HE NPs. F) Comparison of Fourier‐transform infrared (FTIR) spectra between free HE and HE NPs.

The chemical structure of HE is shown in **Figure** **1**A. To enhance its water solubility and blood‐brain barrier (BBB) permeability, HE NPs were prepared using the solvent emulsification‐evaporation method. Scanning electron microscopy (SEM) and transmission electron microscopy (TEM) analyses revealed that HE NPs exhibited a rod‐like morphology, with a diameter of approximately 80 nm and a length of about 800 nm (Figure 1B,C). The zeta potential of HE NPs, determined from electrophoretic light scattering (ELS) measurements, was −8.86 ± 3.77 mV (Figure 1D). Dynamic light scattering (DLS) measurements indicated hydrodynamic diameters of 366.5 ± 162.90 nm and a polydispersity index (PdI) of 0.193 for HE NPs (Figure S1A, Supporting Information). Given the rod‐like morphology of HE NPs, the hydrodynamic diameter does not reflect their true geometric dimensions. Nevertheless, DLS provides a valid metric for assessing colloidal stability. We therefore monitored changes in hydrodynamic diameter and PdI in PBS over 1, 3, and 6 d. Both parameters remained stable throughout this period (Figure S1B, Supporting Information), confirming the colloidal stability of HE NPs for at least 6 d. X‐ray diffraction (XRD) analysis demonstrated that HE monomers displayed sharp characteristic diffraction peaks at 12.24°, 13.72°, 15.58°, 19.62°, 22.56°, 21.44° and 24.84° (2*θ*), indicating high crystallinity (Figure 1E). In contrast, the diffraction patterns of HE NPs were smoother, suggesting a reduction in crystallinity. Fourier‐transform infrared (FTIR) analysis (Figure 1F) revealed that free HE exhibited a strong and broad ─OH stretching vibration peak at 3477 cm^−1^, a sharp peak at 1650 cm^−1^ corresponding to C═O stretching. Compared with HE monomers, the characteristic absorption peaks of HE NPs showed no significant changes in position, likely due to their self‐assembly driven by intermolecular van der Waals forces and synergistic interactions among multiple functional groups.

### 2.2 Biological Distribution and Pharmacokinetics of HE NPs

**Figure 2:**
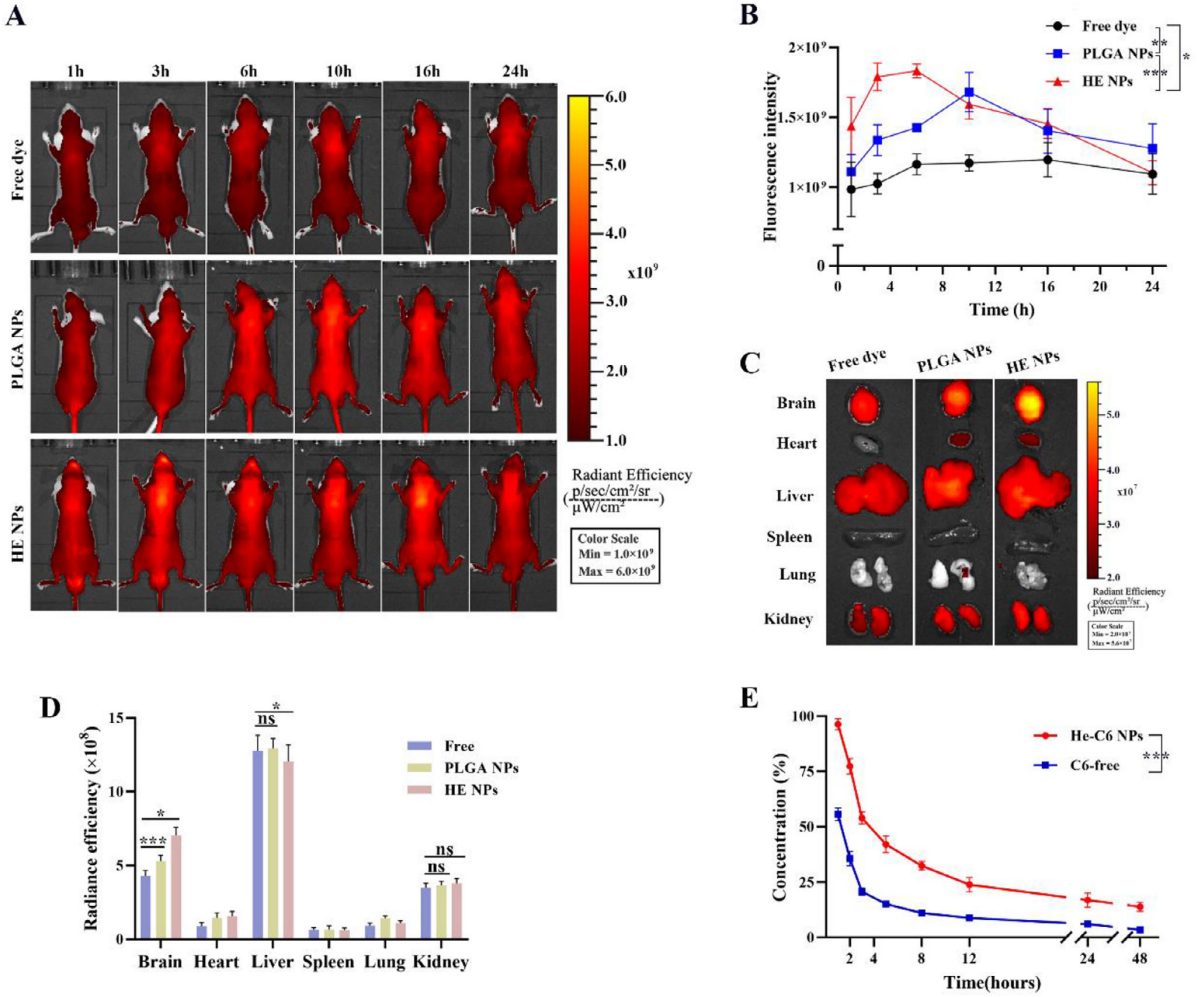
Biodistribution and pharmacokinetics of HE NPs. A) Representative real‐time fluorescence images of nude mice at designated time points post‐intravenous injection of free IR780, PLGA‐IR780 NPs, or HE‐IR780 NPs. B) Quantitative analysis of fluorescence intensity specifically in the brain region derived from whole‐body images. C,D) Ex vivo fluorescence imaging of harvested organs (brain, heart, liver, spleen, lungs, and kidneys) using an IVIS system, with quantification of fluorescence intensity. E) Plasma concentration profile (C6 fluorescence intensity) of HE‐C6 NPs or C6‐free after tail vein injection over time. Data are presented as mean ± standard deviation (SD). *n*  =  3 per group. ns, not significant; *P < 0.05; ***P < 0.001. A *P*‐value < 0.05 was considered statistically significant.

Poly (lactic‐co‐glycolic acid) (PLGA) is a biodegradable polymer with excellent biocompatibility, widely used in the development of nanoparticles for various biomedical applications. The in vivo biodistribution of HE NPs was investigated in healthy nude mice by intravenous administration. Real‐time images of biodistribution were acquired at designated time points post‐injection for free IR780, PLGA‐IR780 NPs, and HE‐IR780 NPs. Results demonstrated that HE‐IR780 NPs exhibited higher fluorescence intensity than free IR780 over 24 h and surpassed PLGA‐IR780 NPs within the first 8 h post‐administration (**Figure** **2**A,B). In DIO mice, free dye, HE‐C6 NPs, or C6‐loaded PLGA‐C6 NPs were administered via tail vein injection. Eight hours post‐injection, mice were euthanized, and brain tissues along with major organs were collected for IVIS imaging. The results demonstrated that, compared to PLGA‐C6 NPs and free C6, HE‐C6 NPs exhibited significantly enhanced signals in the mouse brain, indicating their ability to effectively cross the BBB and accumulate in brain tissues (Figure 2C,D). To further investigate pharmacokinetics, HE‐C6 NPs were injected into mice via the tail vein. Blood samples were collected at different time points, and the blood concentration of HE‐C6 NPs was determined by measuring C6 fluorescence. Figure 2E shows that the fluorescence intensity in the blood decreased rapidly by approximately 50% within 4 h post‐injection of HE‐C6 NPs. In the subsequent time period, compared with C6‐free, HE‐C6 NPs exhibited a longer systemic circulation retention time, indicating excellent stability in the bloodstream.

### 2.3 HE NPs Reduce Body Weight in DIO Mice

**Figure 3:**
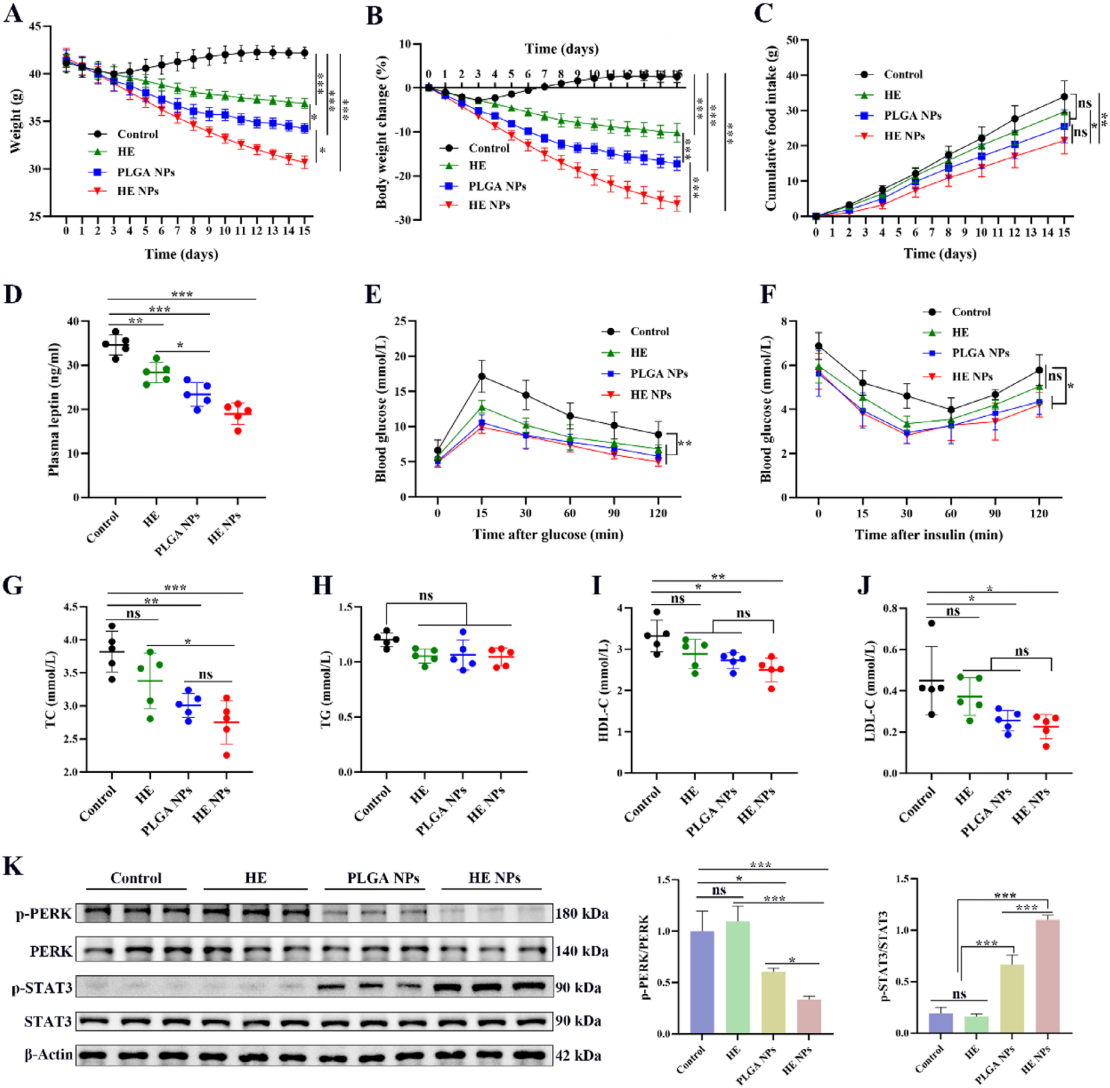
Therapeutic effects of HE NPs on DIO mice. A–C) DIO mice were treated with either control solvent, HE, PLGA NPs, or HE NPs for 14 d. Changes in A) body weight, B) percentage body weight change, and C) cumulative food intake are shown (*n* = 5 per group). D) Plasma leptin levels (*n* = 5 per group). E) Glucose tolerance test (GTT) and F) insulin tolerance test (ITT) curves (*n* = 5 per group). G–J) Plasma lipid profiles: total cholesterol (TC), triglycerides (TG), high‐density lipoprotein cholesterol (HDL‐C), and low‐density lipoprotein cholesterol (LDL‐C) (*n* = 5 per group). K) Representative Western blot images and quantification of hypothalamic p‐PERK/total PERK and p‐STAT3/total STAT3 ratios (*n* = 3 per group). Data are presented as mean ± standard deviation (SD). *P < 0.05, **P < 0.01, ***P < 0.001. A *P*‐value < 0.05 was considered statistically significant.

DIO mice were treated for 14 d with control solvent, HE, PLGA NPs, or HE NPs. Among all groups, HE NPs exhibited the most pronounced reduction in body weight (>20%, *P* < 0.001), significantly outperforming other treatments (**Figure** **3**A,B). Food intake was markedly lower in the HE NPs group compared to the control(*P* < 0.01) and HE groups (*P* < 0.05), though no significant difference was observed versus PLGA NPs (*P* > 0.05) (Figure 3C). Additionally, HE NPs substantially decreased plasma leptin levels (34.6 ± 2.0 ng mL^−1^ vs 19.7 ± 3.2 ng mL^−1^, *P* < 0.001) (Figure 3D). Consistently, food efficiency ratio (FER) analysis showed a significantly lower FER in the HE NPs group compared with controls (Figure S3A, Supporting Information), and leptin‐to‐food ratio (LFR) analysis revealed a more negative LFR in HE NPs‐treated mice (Figure S3B, Supporting Information), suggesting that the reductions in body weight and leptin were not solely due to decreased food intake.

In metabolic assessments, both glucose tolerance (GTT) and insulin sensitivity (ITT) improved in all treatment groups relative to controls, with HE NPs and PLGA NPs showing the strongest enhancements (Figure 3E,F). Lipid profiling revealed that HE NPs significantly reduced total cholesterol (TC), high‐density lipoprotein cholesterol (HDL‐C), and low‐density lipoprotein cholesterol (LDL‐C) (*P* < 0.05) but had no effect on triglycerides (TG) (Figure 3G–J). Western blot (WB) analysis (Figure 3K) indicated that HE NPs and PLGA NPs suppressed hypothalamic PERK phosphorylation (suggesting attenuated ER stress) while elevating STAT3 phosphorylation, indicative of leptin receptor pathway activation.

To evaluate potential toxicity, serum biomarkers of liver (ALT, AST) and kidney (UR, CR) function were analyzed. No adverse effects were observed in any treatment group; instead, HE NPs reduced these parameters versus controls (Figure S2A–D, Supporting Information), confirming that weight loss resulted from pharmacological activity rather than toxicity. Histological examination (H&E staining) demonstrated that HE NPs ameliorated high‐fat‐diet‐induced hepatic steatosis without causing structural damage to the heart, spleen, or kidneys (Figure S2E, Supporting Information). Collectively, HE NPs surpassed HE and PLGA NPs in weight reduction, leptin modulation, and pathway activation, demonstrating superior therapeutic efficacy. Thus, subsequent studies focused exclusively on controls and HE NPs.

### 2.4 HE NPs Have No Effect on Lean Mice

**Figure 4:**
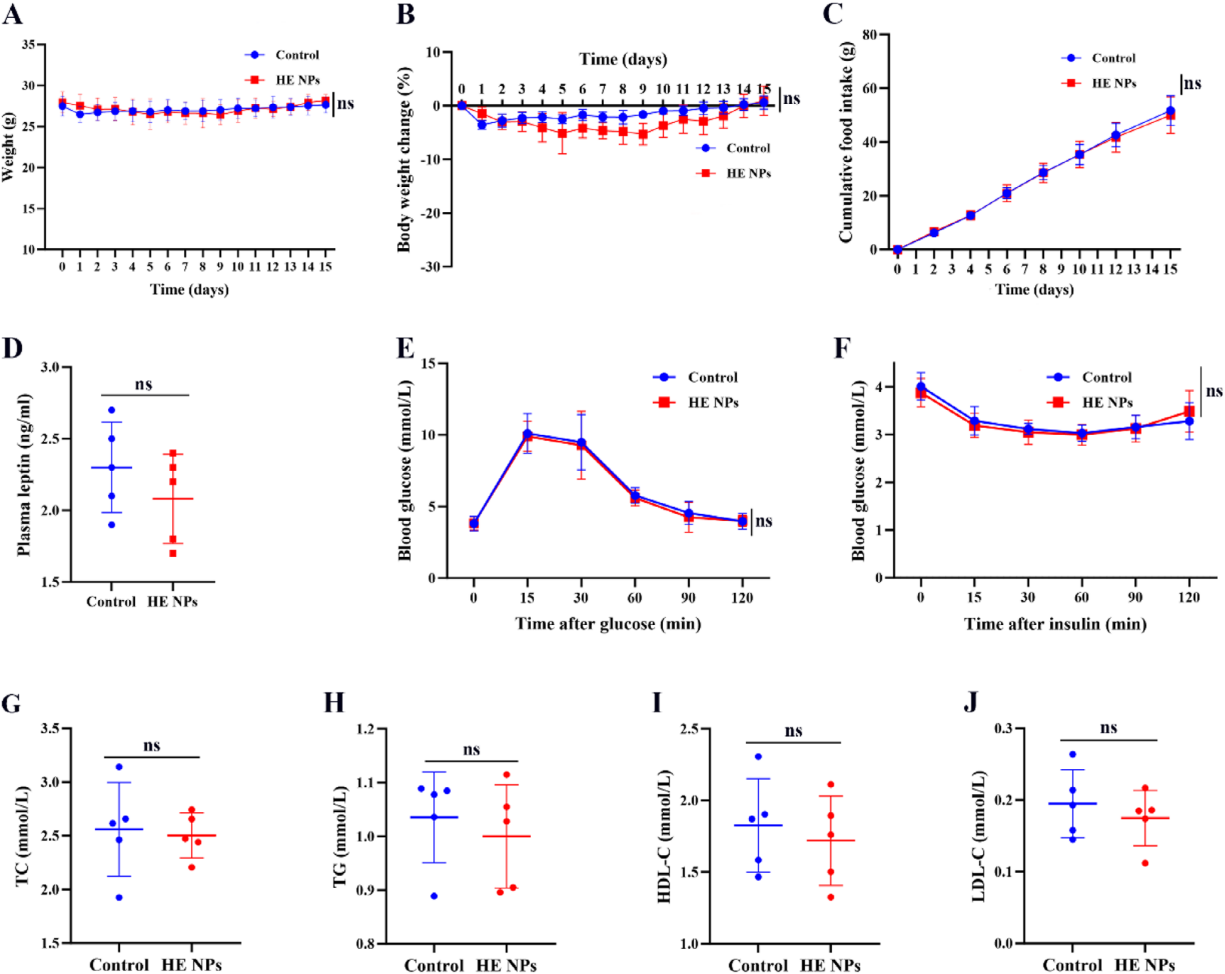
Therapeutic effects of HE NPs on lean mice. A,B) Lean mice were treated with either control solvent or HE NPs for 14 d. Changes in A) body weight and B) percentage body weight change are shown (control solvent, *n* = 5; HE NPs, *n* = 5). C) Cumulative food intake over the treatment period. D) Plasma leptin levels measured at the end of the treatment. E) Glucose tolerance test (GTT) measuring blood glucose levels over time after glucose administration. F) Insulin tolerance test (ITT) assessing blood glucose response following insulin injection. G–J) Plasma lipid profiles: total cholesterol (TC), triglycerides (TG), high‐density lipoprotein cholesterol (HDL‐C), and low‐density lipoprotein cholesterol (LDL‐C). Data are presented as mean ± standard deviation (SD). *n* = 5 per group for all panels. ns, not significant. A *P*‐value < 0.05 was considered statistically significant.

To investigate the generalizability of HE NPs' therapeutic effects, Lean mice were subjected to the same treatment regimen. The results showed that neither control solvent nor HE NPs treatment significantly altered the body weight of Lean mice (**Figure** **4**A), with only minor fluctuations and no statistical differences between groups (Figure 4B). Cumulative food intake (Figure 4C) and plasma leptin levels (Figure 4D) were also comparable. GTT revealed no significant difference in glucose clearance between HE NP‐treated lean mice and controls (Figure 4E), and ITT showed similar insulin sensitivity between the two groups (Figure 4F). Moreover, analysis of lipid metabolism‐related markers demonstrated that HE NP treatment did not produce statistically significant differences in TC, TG, HDL‐C, or LDL‐C in lean mice compared with controls (Figure 4G–J). Collectively, these results indicate that HE NPs have no notable impact on metabolic parameters in Lean mice.

### 2.5 Effects of HE NPs on ob/ob Mice

**Figure 5:**
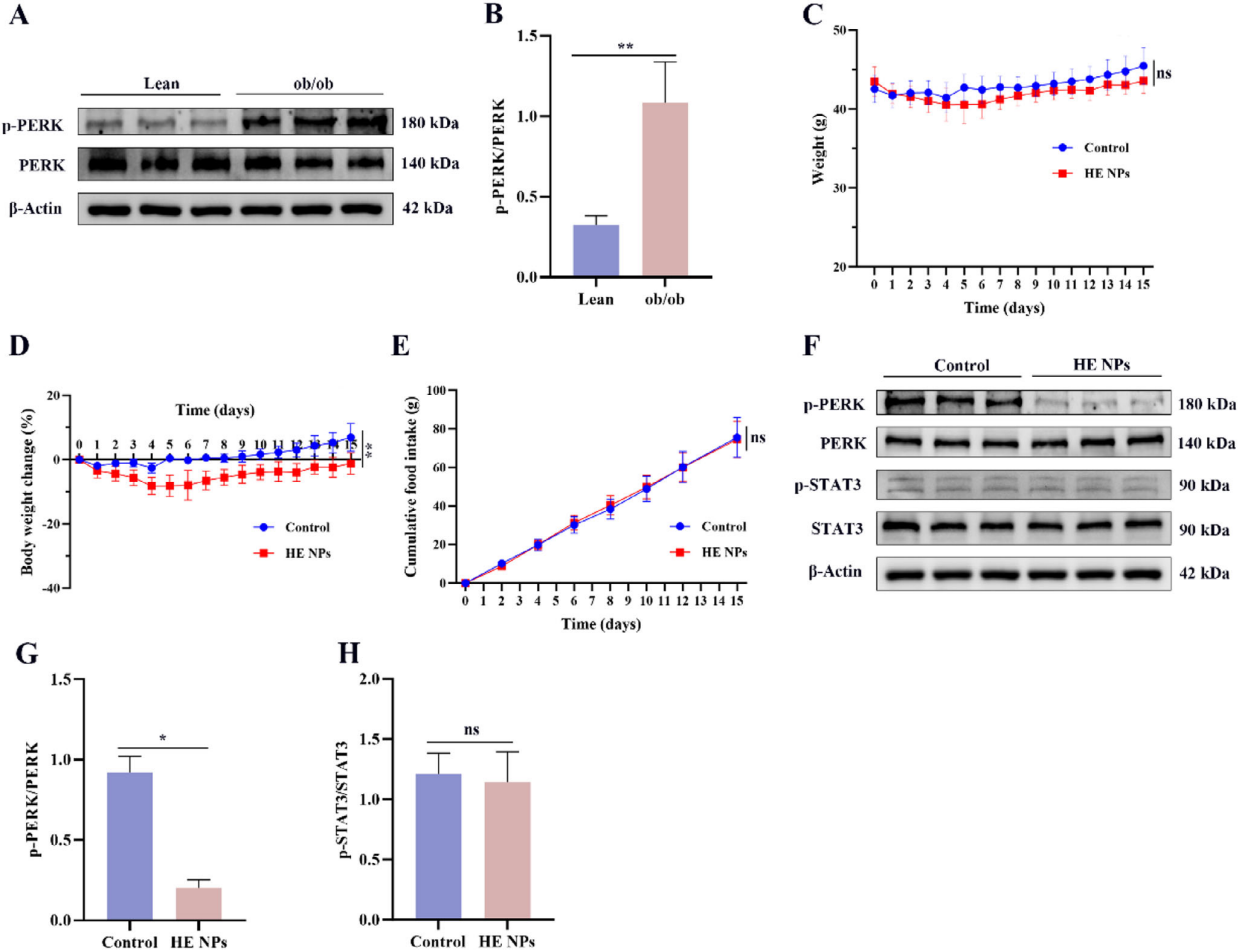
Effects of HE NPs on ob/ob mice. A) Representative Western blot images of p‐PERK and total PERK in the hypothalamus of lean and ob/ob mice. B) Quantified ratio of p‐PERK to total PERK (*n* = 3 per group). C–E) ob/ob mice were treated with either control solvent or HE NPs for 14 d. Changes in C) body weight, D) percentage body weight change, and E) cumulative food intake are shown (*n* = 5 per group). F) Representative Western blot images of p‐PERK, total PERK, p‐STAT3, and total STAT3 in the hypothalamus. G) Quantified ratio of p‐PERK to total PERK (*n* = 3 per group). H) Quantified ratio of p‐STAT3 to total STAT3 (*n* = 3 per group). Data are presented as mean ± standard deviation (SD). *P < 0.05, **P < 0.01, ***P < 0.001. A *P*‐value < 0.05 was considered statistically significant.

Given that the weight‐reducing and appetite‐suppressing effects of HE NPs were observed only in DIO mice, we further investigated their therapeutic efficacy in leptin‐deficient ob/ob mice to elucidate the underlying mechanisms. First, we found that hypothalamic PERK phosphorylation levels in ob/ob mice were significantly higher than those in Lean mice (**Figure** **5**A,B), indicating elevated hypothalamic ER stress. Body weight monitoring revealed that HE NPs treatment did not significantly reduce the absolute body weight of ob/ob mice (*P* > 0.05) (Figure 5C), whereas the percentage change in body weight was significantly lower in the HE NPs group compared with controls (*P* < 0.01) (Figure 5D), indicating a modest attenuation of weight gain. Cumulative food intake did not differ significantly between groups (*P* > 0.05) (Figure 5E). Western blot results showed that HE NPs reduced hypothalamic PERK phosphorylation in ob/ob mice but had no significant effect on STAT3 phosphorylation (Figure 5F–H).

**Figure 6:**
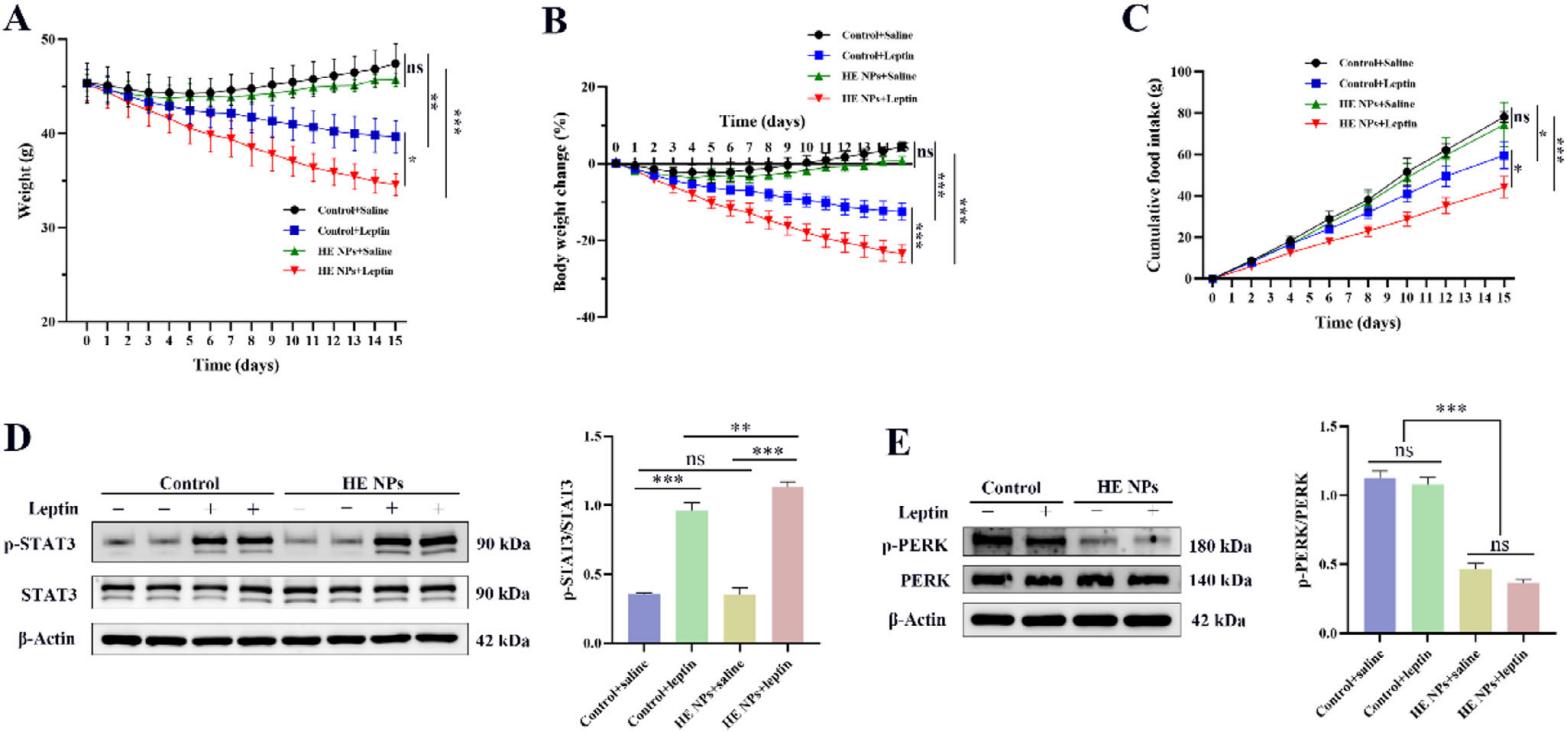
Leptin‐sensitizing effects of HE NPs in ob/ob mice. A–C) ob/ob mice pretreated with either control solvent or HE NPs were further treated with saline or leptin. Changes in A) body weight, B) percentage body weight change, and C) cumulative food intake are shown (*n* = 5 per group). D) Representative Western blot images of p‐STAT3 and total STAT3 in the hypothalamus, along with the quantified ratio of p‐STAT3 to total STAT3 (*n* = 3 per group). E) Representative Western blot images of p‐PERK and total PERK in the hypothalamus, along with the quantified ratio of p‐PERK to total PERK (*n* = 3 per group). Data are presented as mean ± standard deviation (SD). ns, not significant; *P < 0.05, **P < 0.01, ***P < 0.001. A *P*‐value < 0.05 was considered statistically significant.

To explore the relationship between HE NPs' therapeutic effects and leptin, ob/ob mice were divided into control solvent or HE NPs pretreatment groups, with each subgroup further treated with either saline or leptin. Compared to the control solvent + saline group, leptin treatment significantly reduced body weight and food intake in ob/ob mice, and the combination of HE NPs with leptin (HE NPs + leptin) further enhanced these effects (**Figure** **6**A–C). In the control solvent pretreatment group, leptin treatment increased hypothalamic STAT3 phosphorylation without affecting PERK phosphorylation, whereas the combination of HE NPs and leptin not only further increased STAT3 phosphorylation but also significantly reduced PERK phosphorylation levels (Figure 6D,E). These results suggest that HE NPs possess leptin‐sensitizing properties.

### 2.6 HE NPs Act as a Leptin Sensitizer in DIO Mice

**Figure 7:**
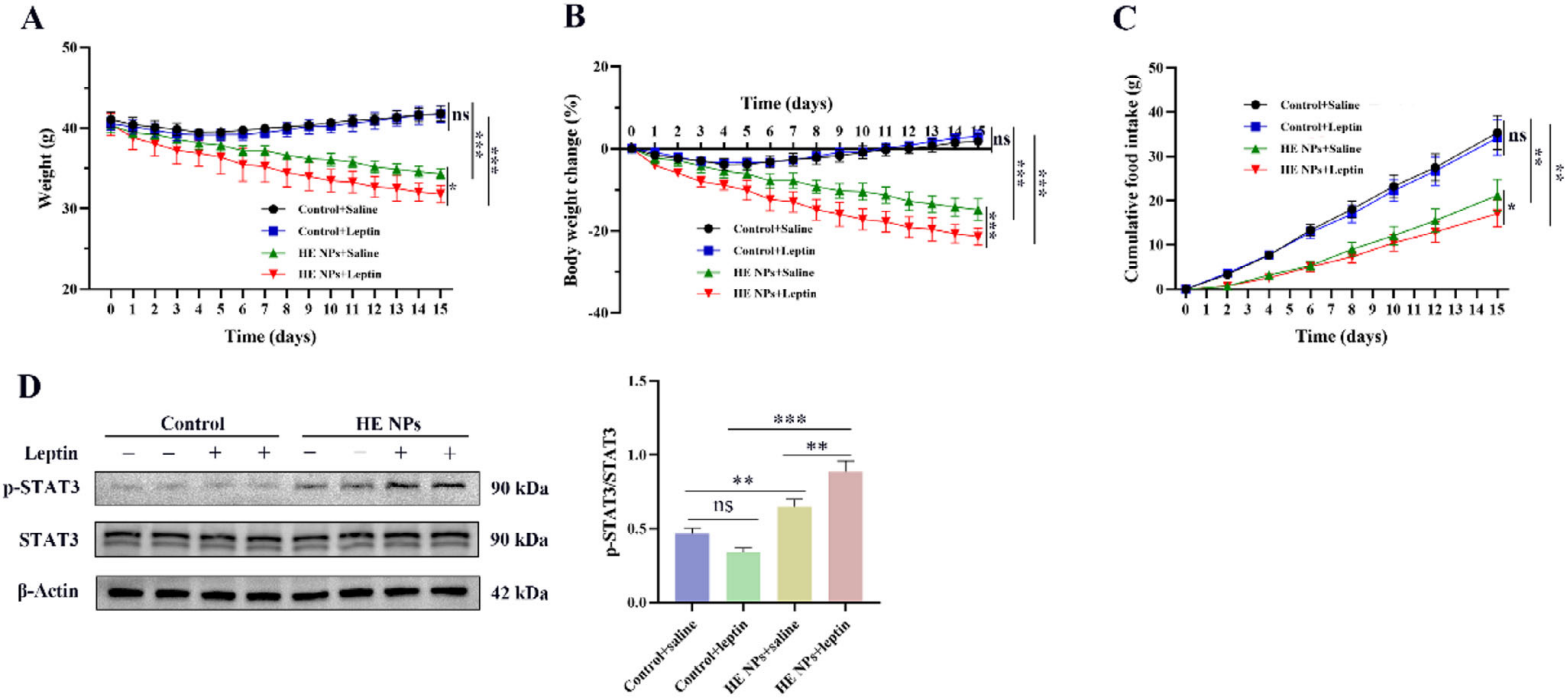
Leptin‐sensitizing effects of HE NPs in DIO mice. A–C) DIO mice pretreated with either control solvent or HE NPs were further treated with saline or leptin. Changes in A) body weight, B) percentage body weight change, and C) cumulative food intake are shown (*n* = 5 per group). D) Representative Western blot images of p‐STAT3 and total STAT3 in the hypothalamus, along with the quantified ratio of p‐STAT3 to total STAT3 (*n* = 3 per group). Data are presented as mean ± standard deviation (SD). ns, not significant; *P < 0.05, **P < 0.01, ***P < 0.001. A *P*‐value < 0.05 was considered statistically significant.

To investigate the leptin‐modulating effects of HE NPs, we tested the impact of combined HE NPs and leptin treatment in DIO mice. DIO mice were pretreated with either control solvent or HE NPs, and each subgroup was then treated with saline or leptin. The results showed that, compared to the control solvent + saline group, leptin treatment alone had no significant effect on body weight or food intake in DIO mice (*P* > 0.05) (**Figure** **7**A–C), confirming leptin resistance in DIO mice. However, HE NPs + saline treatment significantly reduced body weight and food intake in DIO mice, and HE NPs pretreatment markedly enhanced leptin sensitivity, as evidenced by further reductions in body weight and food intake in the HE NPs + leptin group (Figure 7A–C). Western blot analysis revealed that, in DIO mice, control solvent + saline and control solvent + leptin treatments had no significant effect on STAT3 phosphorylation, whereas HE NPs + saline significantly increased basal STAT3 phosphorylation levels (Figure 7D). The HE NPs + leptin treatment further enhanced STAT3 phosphorylation, significantly surpassing all other groups (Figure 7D), indicating that HE NPs enhance leptin signaling and ameliorate leptin resistance.

### 2.7 HE NPs Ameliorate Sleep‐Disordered Breathing in DIO Mice

**Figure 8:**
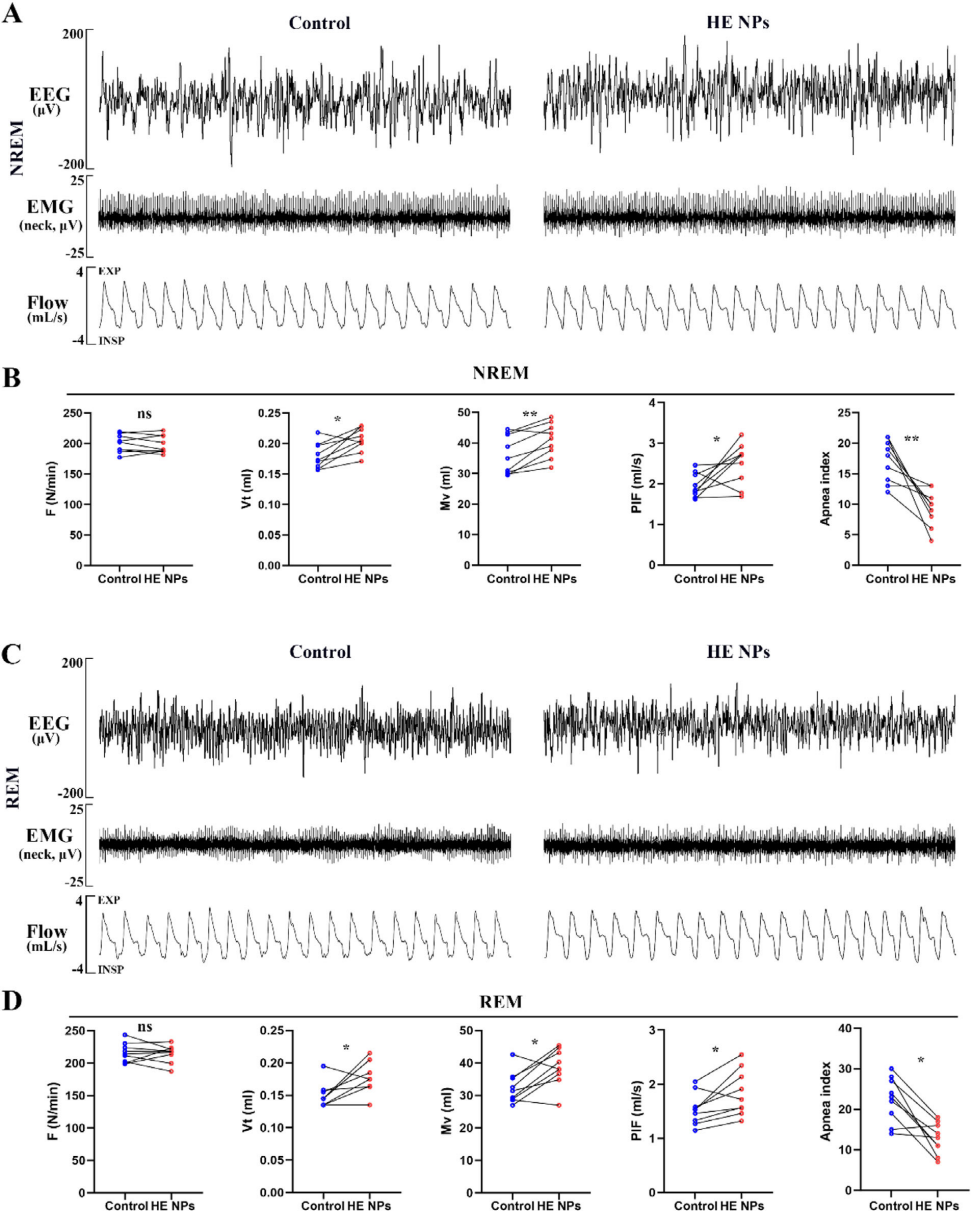
Effects of HE NPs on sleep and respiration in DIO mice. A) Representative electroencephalogram (EEG), neck electromyogram (EMG), and respiratory waveforms (top to bottom) during NREM sleep after treatment with control solvent (left) or HE NPs (right). B) Respiratory parameters during NREM sleep after treatment with control solvent or HE NPs, including respiratory frequency (F), tidal volume (Vt), minute ventilation (Mv), peak inspiratory flow (PIF), and apnea index (AI). C) Representative EEG, EMG, and respiratory waveforms (top to bottom) during REM sleep after treatment with control solvent (left) or HE NPs (right). D) Respiratory parameters during REM sleep after treatment with control solvent or HE NPs, including respiratory frequency (F), tidal volume (Vt), minute ventilation (Mv), peak inspiratory flow (PIF), and apnea index (AI). *n* = 9 per group. ns, not significant; *P < 0.05, **P < 0.01, ***P < 0.001. A *P*‐value < 0.05 was considered statistically significant.

Previous studies have demonstrated the feasibility of DIO mice as a model for SDB.^[^ ^23^ ^]^ To further evaluate the impact of HE NPs on respiratory function during sleep, we analyzed respiratory parameters in DIO mice during both NREM and REM. During NREM sleep, HE NPs treatment significantly improved inspiratory flow limitation in DIO mice (**Figure** **8**A). While respiratory frequency showed no significant difference, tidal volume (Vt), minute ventilation (Mv), and peak inspiratory flow (PIF) were significantly increased (Figure 8B). Additionally, the apnea index (AI) decreased significantly from 17.1 ± 3.5 events h^−1^ in the control group to 9.3 ± 3.0 events h^−1^ in the HE NPs‐treated group (*P* < 0.01) (Figure 8B). During REM sleep, HE NPs also demonstrated significant improvements in respiratory function, with increased tidal volume, minute ventilation, and PIF, and a reduction in AI from 22.4 ± 5.6 events h^−1^ in the control group to 12.8 ± 3.9 events h^−1^ (*P* < 0.05) (Figure 8C,D). These findings indicate that HE NPs effectively improve respiratory function in DIO mice during both NREM and REM sleep.

## 3 Discussion

This study elucidates the multifaceted mechanisms and potential therapeutic applications of hesperidin‐based self‐assembled nanoparticles (HE NPs) in treating obesity‐related SDB. The findings demonstrate that HE NPs significantly ameliorate leptin resistance in DIO mice by alleviating hypothalamic ER stress and enhancing STAT3 phosphorylation. This discovery provides new insights into the molecular mechanisms underlying leptin resistance and establishes a theoretical foundation for developing ER stress‐targeted therapeutic strategies.

Hesperidin, a natural phenolic compound, has garnered attention for its potent antioxidant and anti‐inflammatory properties.^[^ ^24^ ^]^ However, its poor water solubility and bioavailability have severely limited its clinical application.^[^ ^25^ ^]^ To overcome these limitations, this study innovatively developed self‐assembled HE NPs. Experimental results revealed that, compared to PLGA‐C6 NPs and C6‐free, HE‐C6 NPs exhibited superior BBB penetration, effectively delivering the drug to brain tissues. Notably, the self‐assembly preparation method employed in this study avoided the introduction of exogenous compounds, ensuring the biocompatibility of the delivery system. Pharmacokinetic studies further demonstrated that intravenous injection of HE‐C6 NPs resulted in a biphasic elimination profile: an initial rapid decline in blood fluorescence within 4 h indicated efficient distribution to target tissues, while a subsequent slow elimination rate confirmed prolonged systemic circulation. This unique pharmacokinetic behavior may be attributed to the distinctive morphology of HE NPs. Research suggests that the geometric shape of nanoparticles, particularly the aspect ratio, significantly influences their interactions with biological systems. The rod‐like structure of HE NPs may reduce macrophage phagocytosis by limiting contact with cell surfaces while enhancing their ability to cross biological barriers, thereby optimizing drug delivery.^[^ ^26^ ^]^

In terms of pharmacological effects, HE NPs significantly reduced food intake and body weight in DIO mice while modulating the expression of key signaling molecules such as P‐PERK and P‐STAT3. The therapeutic efficacy of HE NPs exhibited leptin dependency: no significant effects were observed in lean mice with low leptin levels, whereas in leptin‐deficient ob/ob mice, HE NPs had limited effects on absolute body weight but significantly reduced hypothalamic ER stress markers. To further validate this hypothesis, leptin combination therapy experiments in ob/ob and DIO mice showed that leptin alone reduced food intake and body weight in ob/ob mice, while the combination of HE NPs and leptin produced a synergistic effect. In DIO mice, the therapeutic effects of HE NPs were further amplified when combined with leptin, whereas leptin alone showed no significant difference compared to saline treatment. This confirmed that HE NPs act as leptin sensitizers, with their efficacy dependent on the presence of high leptin levels, whether endogenous or exogenous.

Under conditions of exogenous leptin deficiency but preserved endogenous leptin, HE NPs alone were able to enhance the basal phosphorylation level of hypothalamic STAT3, suggesting that their effect does not entirely depend on exogenous leptin supplementation, but may be achieved by potentiating endogenous leptin signaling. Importantly, the STAT3 activation induced by HE NPs was primarily mediated through leptin receptor‐dependent pathways, rather than direct activation of STAT3 by hesperidin. In contrast, previous studies have shown that hesperidin derivatives, such as 7,3′‐dimethoxy hesperetin (DMHP), can inhibit excessive JAK2‐STAT3 activation under pathological conditions such as inflammatory arthritis,^[^ ^27^ ^]^ indicating a bidirectional regulatory potential of hesperidin compounds on STAT3 depending on tissue type and pathological context. In the DIO model used in this study, basal hypothalamic STAT3 activity was low and leptin responsiveness was impaired, representing a signaling hyporesponsive state. Consequently, the primary action of HE NPs in this context was to enhance leptin signal transduction efficiency rather than suppress its activation. Consistently, HE NPs markedly reduced hypothalamic ER stress markers in both DIO and ob/ob mice, whereas exogenous leptin alone showed no significant improvement, further supporting a mechanism whereby HE NPs enhance leptin sensitivity by alleviating ER stress.

Regarding the mechanisms underlying improvements in the leptin system, the results suggest that HE NPs may act through two complementary but mechanistically distinct pathways. First, prior research has indicated that hesperidin can bind with high affinity to leptin receptors and induce conformational changes, thereby enhancing leptin‐receptor interactions and directly activating downstream signaling pathways.^[^ ^28^ ^]^ This mechanism may contribute to the alleviation of leptin resistance by overcoming receptor‐binding defects. Second, our data indicate that HE NPs can mitigate hypothalamic ER stress and improve signal transduction efficiency, thereby enhancing responsiveness to endogenous leptin and effectively increasing leptin sensitivity. This “signal amplification” effect does not require exogenous leptin supplementation but is achieved through optimization of endogenous leptin signaling. Furthermore, previous reports in ovariectomized rat models have shown that hesperidin‐related compounds can reduce circulating leptin levels and improve adipose tissue function,^[^ ^29^ ^]^ highlighting their multi‐faceted regulatory potential within the leptin system. Taken together, HE NPs likely exert synergistic effects via both “receptor modulation” and “signal amplification” mechanisms, improving leptin system function at multiple levels.

This study also found that HE NPs effectively alleviated upper airway obstruction during sleep in leptin‐resistant DIO mice, improved inspiratory flow limitation, and significantly reduced apnea events during both NREM and REM sleep, suggesting direct or indirect modulation of respiratory and respiratory pump muscle control. Although the precise mechanisms remain unclear, existing research indicates that leptin activates neurons in respiratory‐related brain regions, including key central chemosensitive areas such as the rostral ventrolateral medulla (RVLM).^[^ ^30^, ^31^ ^]^ Leptin may enhance upper airway function by increasing sensitivity to carbon dioxide, thereby promoting hypoglossal motor neuron and genioglossus muscle activity or directly stimulating hypoglossal motor neurons.^[^ ^32^ ^]^ Furthermore, transneuronal tracing experiments have confirmed synaptic connections between leptin receptor‐positive cells and respiratory motor neurons.^[^ ^14^ ^]^ In this study, HE NPs treatment significantly increased STAT3 phosphorylation levels in the brain, suggesting that their therapeutic effects on SDB may be related to these mechanisms. Additionally, the regulation of upper airway dilator muscle tone involves complex neurotransmitter systems: during the transition from wakefulness to NREM sleep, reduced norepinephrine levels in the hypoglossal motor nucleus (HMN) are the primary cause of decreased upper airway dilator muscle tone,^[^ ^33^ ^]^ while enhanced cholinergic system activity (mediated by muscarinic M2 receptors) dominates during REM sleep.^[^ ^34^ ^]^ Given that hesperidin has been shown to modulate norepinephrine and cholinergic systems,^[^ ^35^, ^36^, ^37^ ^]^ this provides new directions for investigating the mechanisms by which HE NPs improve SDB.

In terms of safety evaluation, two weeks of HE NPs treatment in DIO mice did not induce significant pathological changes in major organs, confirming their favorable safety profile. Previous studies also support this conclusion: hesperidin‐loaded gold nanoparticles exhibited potent anti‐inflammatory effects without observable toxicity or adverse behavioral reactions, and no macroscopic organ changes were noted at the end of treatment.^[^ ^38^ ^]^ The advantages of hesperidin therapy include its high safety, non‐accumulative nature, and limited side effects even during pregnancy, with no mutagenic, toxic, or carcinogenic effects.^[^ ^39^ ^]^ Clinical data indicate that the incidence of mild side effects from oral hesperidin (approximately 10%) is even lower than that of the placebo group (13.9%).^[^ ^40^ ^]^ However, potential drug interactions, such as increased absorption of vincristine when co‐administered with hesperidin, as well as interactions with daunorubicin, should be noted.^[^ ^39^ ^]^

In summary, this study demonstrates that HE NPs significantly reduce body weight and suppress food intake in DIO mice by enhancing leptin sensitivity and alleviating SDB, primarily through hypothalamic ER stress reduction and leptin signaling restoration. Despite promising efficacy and safety, limitations include the exclusive use of male DIO mice (due to sex‐specific metabolic differences), reliance on intravenous administration, and incomplete mechanistic insights into ER stress modulation. Future studies should explore chronic dosing, alternative delivery routes (e.g., intranasal), and HE NPs’ effects on neuronal function and upper airway muscle regulation to further elucidate therapeutic mechanisms. These findings position HE NPs as a novel leptin‐sensitizing strategy for obesity and SDB.

## 4 Experimental Section

### Experimental Animals

Male C57BL/6J and ob/ob mice, along with their respective diets, were purchased from Shulaibao Biotechnology Co., Ltd. (Wuhan, China). The following dietary interventions were implemented: 6 week old C57BL/6J mice were divided into two groups—the diet‐induced obesity (DIO) group, fed a high‐fat diet (TD 0 3584, 58.4% fat‐derived calories), and the Lean group, fed a standard diet (13% fat‐derived calories). The ob/ob mice were also maintained on a standard diet. All mice were housed under a 12 h light‐dark cycle (light period: 9:00‐21:00) with free access to food and water. This study strictly adhered to the National Institutes of Health (NIH) guidelines for the care and use of laboratory animals, and all experimental protocols were approved by the Animal Ethics Committee of Renmin Hospital of Wuhan University.

### Drugs and Antibodies

Hesperidin (HE) was purchased from MedChemExpress (purity ≥98%, CAS No.: 520‐26‐3, USA). The following antibodies were used: Anti‐Phospho‐PERK (3179, Cell Signaling Technology), Anti‐PERK (5683, Cell Signaling Technology), Anti‐p‐STAT3 (ab76315, Abcam), Anti‐STAT3 (ab68153, Abcam), and Anti‐β‐Actin (GB15001, Servicebio).

### Synthesis of HE NPs

10 mg HE was dissolved in 1 mL of organic solvent ((ethyl acetate: dimethyl sulfoxide = 19:1). After complete dissolution, the solution was added dropwise to 3 mL of vigorously stirred 2.5% Pluronic F‐127 (F127) solution. The mixture was placed on ice and sonicated at 120 W for 11 min and 30 s (10 s on/5 s off). The resulting solution was then poured into 30 mL of 0.3% F127 solution and stirred overnight at room temperature. The next day, the mixture was centrifuged at 18000 rpm for 30 min, and the supernatant was discarded. The precipitate was resuspended in 1 mL of distilled water and centrifuged at 1000 rpm for 5 min to remove the precipitate and stored at 4 °C until use. Using identical methodology, coumarin‐6‐loaded HE NPs and IR780‐incorporated HE NPs were prepared by co‐dissolving each fluorescent marker with HE in the organic phase.

### Scanning Electron Microscopy (SEM)

HE NPs samples at different dilution ratios were evenly deposited onto silicon wafers and dried at room temperature to remove the solvent. After drying, the samples were sputter‐coated with gold under vacuum and argon gas at a current of 30 mA for 60 s to form a uniform conductive gold layer. The samples were then mounted on SEM stubs and observed using a GeminiSEM 500 field‐emission scanning electron microscope (Carl Zeiss AG, Germany). The acceleration voltage was set to 5 kV, and images were captured at a magnification of 10000× with optimized parameters for clarity.

### Transmission Electron Microscopy (TEM)

Dried HE NPs were suspended in ultrapure water and adjusted to a concentration of 50 µg mL^−1^ with uniform dispersion. A droplet of the suspension was placed on a porous carbon‐coated copper grid and allowed to stand for 5 min. Excess liquid was removed using filter paper, and the grid was air‐dried in a fume hood. The dried grid was mounted on a TEM sample holder and observed using a Hitachi HT‐7700 transmission electron microscope (Hitachi High‐Tech, Japan) at an acceleration voltage of 100 kV to obtain high‐resolution images.

### Zeta Potential and Hydrated Particle Size

The freeze‐dried HE NPs powder was diluted to a 1 mg mL^−1^ aqueous solution. Zeta potential and hydrated particle size were measured using electrophoretic light scattering (ELS) and dynamic light scattering (DLS), respectively, with a Zetasizer Nano ZSP (Malvern Instruments Ltd., UK).

### Fourier Transform Infrared Spectroscopy (FTIR)

Five milligrams of HE or HE NPs freeze‐dried powder were weighed and mixed with an appropriate amount of potassium bromide (KBr) powder. The mixture was thoroughly ground in a mortar and then transferred to a pellet mold. A transparent pellet was prepared under high pressure using a hydraulic press. The prepared KBr pellet was placed in the sample chamber of an FTIR spectrometer (Nicolet iN10, Thermo Fisher Scientific, USA), and the infrared spectrum was recorded over a scanning range of 400–4000 cm^−1^.

### X‐Ray Diffraction (XRD)

XRD analysis was performed on the raw material (HE) and its nanoparticles (HE NPs) using an X‐ray diffractometer (D8 ADVANCE, Bruker, Germany). Fifty milligrams of HE or HE NPs freeze‐dried powder were ground uniformly in an agate mortar and sieved to ensure consistent particle size. The powder was then pressed into a pellet and placed in the X‐ray diffractometer. The scanning range was set from 8° to 80° (2θ), and the XRD diffraction patterns were recorded.

### Pharmacokinetic Analysis

To investigate the pharmacokinetics in mice, HE NPs loaded with coumarin‐6 (HE‐C6 NPs, 20 mg kg^−1^) or an equivalent dose of C6‐Free were administered via tail vein injection (*n* = 3 per group). Blood samples were collected from the tail vein at predetermined time points (1.0, 2.0, 3.0, 5.0, 8.0, 12.0, 24.0, and 48.0 h). The blood samples were centrifuged at 3000 rpm for 10 min to separate blood cells. Methanol was then used to lyse the nanoparticles, and the fluorescence intensity of the samples was measured using a microplate reader. A curve was plotted based on the fluorescence intensity over time to evaluate the pharmacokinetic properties of HE NPs in mice.

### Biodistribution Assessment

Healthy nude mice (*n* = 3 per group) were intravenously injected with free IR780, PLGA‐IR780 NPs, or HE‐IR780 NPs. IR780 fluorescence was assessed at 1, 3, 6, 10, 16, and 24 h post‐injection (p.i.) using an IVIS imaging system. In DIO mice, free C6, HE‐C6 NPs, and standard nanoparticles (PLGA‐C6 NPs) were separately injected into 18 week old DIO mice via the tail vein (*n* = 3 per group). Eight hours post‐injection, the mice were euthanized and subjected to cardiac perfusion to remove blood. Major organs, including the brain, heart, liver, lungs, kidneys, and spleen, were harvested and imaged using an IVIS Lumina XRMS Series III in vivo imaging system. The fluorescence distribution and intensity in the brain and other organs were analyzed using Living Image 4.4 software (Xenogen). This provided preliminary insights into the targeting efficiency and tissue distribution characteristics of HE NPs.

### Chronic Treatment Study of HE NPs: HE NPs Monotherapy Experiment

Twenty‐five DIO mice (6 week old C57 mice fed a high‐fat diet for 8 weeks), were used, of which 5 were randomly selected to measure baseline plasma leptin levels. The remaining 20 mice were randomly divided into four groups (*n* = 5 per group): control (equal volume of solvent, i.v.), HE (100 mg kg^−1^, oral gavage), PLGA NPs (20 mg kg^−1^, i.v.), and HE NPs (20 mg kg^−1^, i.v.), with treatments administered every 2 d for 2 weeks. Food intake and body weight were monitored. At the end of the experiment, blood was collected to measure plasma leptin levels, and hypothalamic tissues were harvested for Western blot (WB) analysis. Ten Lean mice (18 weeks old), and ten ob/ob mice (8 weeks old) were randomly divided into Control (equal volume of solvent) and HE NPs (20 mg kg^−1^) groups, respectively, with the same treatment schedule. In lean mice, plasma leptin levels were measured, while in ob/ob mice, hypothalamic tissues were collected for WB analysis.

### Chronic Treatment Study of HE NPs: HE NPs and Leptin Combination Therapy Experiment

Twenty ob/ob mice (8 weeks old) and twenty DIO mice (fed a high‐fat diet from 6 weeks of age for 8 weeks, 14 weeks old at the start of the experiment) were randomly divided into Control and HE NPs (20 mg kg^−1^, i.v.) groups. Treatment lasted for 6 d (administered every 2 d, totaling 3 doses) to allow adaptation. On day 6, each group was further divided into two subgroups, receiving either intraperitoneal leptin (1 mg kg^−1^) or an equal volume of saline for 2 weeks. This resulted in four groups: control + saline, control + leptin, HE NPs + saline, and HE NPs + Leptin, with 5 mice per group. Food intake and body weight were monitored throughout the experiment, and hypothalamic tissues were collected for WB analysis at the end.

### Acute Treatment Study of HE NPs

A randomized crossover study was designed. Ten 6 week old C57 mice were fed a high‐fat diet for 12 weeks and then randomly divided into two groups (5 mice each), receiving either 20 mg kg^−1^ HE NPs or an equal volume of control solvent. Respiratory and sleep parameters were monitored immediately for 6 h post‐administration. After a 1 week washout period, the treatments were crossed over, and monitoring was repeated for another 6 h. This acute experiment minimized confounding effects from leptin‐induced weight loss. During the monitoring, the electrode of one mouse became dislodged, so data from 9 mice were included in the analysis.

### Plasma Leptin Measurement

Blood (1–2 mL) was collected from the carotid artery of each group and placed in anticoagulant‐containing tubes. After mixing, the blood was centrifuged at 3500 rpm for 15 min at 4 °C, and the plasma supernatant was stored at −80 °C. A mouse leptin ELISA kit was used for detection: standard concentration gradients were prepared according to the manufacturer's instructions, and plasma samples were appropriately diluted. Samples and standards were added to the microplate, followed by incubation, washing, and color development. Absorbance at 450 nm was measured using a microplate reader, and sample concentrations were calculated based on the standard curve.

### Food Efficiency Ratio (FER) and Leptin‐to‐Food Ratio (LFR)

To evaluate the metabolic effects of therapeutic interventions, two key parameters were calculated: the food efficiency ratio (FER) and the leptin‐to‐food ratio (LFR). FER was determined by dividing the body weight change (g) of each animal by its cumulative food intake (g), providing a measure of weight change per unit of food consumed. LFR was calculated by dividing the change in leptin levels (post‐treatment level minus the pretreatment average leptin level of five batch‐matched control mice) by cumulative food intake (g). These metrics help determine whether observed changes in leptin and body weight were solely attributable to alterations in food intake or involved additional mechanisms.

### Glucose Homeostasis and Metabolic Measurements

For glucose tolerance tests (GTT), mice were fasted overnight for 14 h, and baseline blood glucose was measured at time 0 from the tail vein, followed by intraperitoneal injection of glucose (1 g kg^−1^). Blood glucose levels were subsequently determined at 15, 30, 60, 90, and 120 min post‐injection. For insulin tolerance tests (ITT), mice were fasted for 6 h, and baseline glucose was measured at time 0, followed by intraperitoneal injection of recombinant human insulin (1 IU kg^−1^). Blood glucose levels were then measured at the same time intervals as in the GTT. Serum lipid profiles, including total cholesterol (TC), triglycerides (TG), high‐density lipoprotein cholesterol (HDL‐C), and low‐density lipoprotein cholesterol (LDL‐C), as well as liver function (alanine aminotransferase, ALT; aspartate aminotransferase, AST) and kidney function (urea, UR; creatinine, CR), were measured using commercial assay kits.

### Western Blot

Tissue samples stored at −80 °C were homogenized in pre‐chilled lysis buffer and incubated on ice for 30 min. After sonication, the lysates were centrifuged at 12 000 rpm for 15 min at 4 °C, and the supernatant was collected. Protein concentration was determined using the BCA protein assay. Lysates were heated at 100 °C for 5 min and mixed with loading buffer. Proteins (30 µg) were separated by SDS‐PAGE and transferred to PVDF membranes (0.45 µm, Millipore). Membranes were blocked with 5% skim milk for 1 h, incubated with primary antibodies at 4 °C overnight, washed with TBST, and then incubated with HRP‐conjugated secondary antibodies at room temperature for 1 h. ECL substrate was applied for 30 s in the dark, and chemiluminescence was detected using an imaging system. Band intensity was quantified using ImageJ.

### Hematoxylin and Eosin Staining

Mice were anesthetized with isoflurane and perfused via the heart. The heart, liver, kidneys, and spleen were harvested and fixed in 4% paraformaldehyde for 24 h. Tissues were dehydrated in graded ethanol, cleared in xylene, embedded in paraffin, and sectioned at 4 µm. Sections were deparaffinized, rehydrated, stained with hematoxylin for 3 min, differentiated in acid ethanol for 2–5 s, blued in bluing solution for 2–5 s, and counterstained with eosin for 3 min. After dehydration and clearing, sections were mounted with neutral resin. Images were visualized using an Olympus BX51 microscope.

### Sleep Monitoring

Electrode implantation and sleep data analysis were performed as previously described.^[^ ^41^ ^]^ Briefly, mice anesthetized with 1–2% isoflurane were fixed in a stereotaxic frame. After disinfection, a midline incision was made to expose the skull, and the periosteum was removed. EEG/EMG head‐mounted electrodes (Pinnacle Technology, Lawrence, KS) were placed on the skull surface, and screw holes were drilled and secured (0.10 in. screws for anterior holes, 0.12 in. screws for posterior holes). Two EMG electrodes were inserted subcutaneously into the neck muscles. The incision was sutured, and the head mount was sealed with dental cement. Postoperative analgesia (buprenorphine) was administered for at least 3 d, and mice were allowed to recover for 5–7 d before monitoring. Awake mice were placed in recording chambers for 6 h (10:00 AM to 4:00 PM). Sleep scoring was performed using Sirenia Sleep Pro (Pinnacle Technology) and two independent blinded evaluators.

### Respiratory Monitoring

Whole‐body plethysmography (WBP, Shanghai Tawang Intelligent Technology Co., Ltd., Shanghai, China) was used to monitor respiratory parameters. Mice were acclimated to the chamber for 3 d before the experiment. During the experiment, awake mice were placed in the WBP chamber for 6 h (10:00 AM to 4:00 PM), synchronized with sleep recording. A 30‐minute stabilization period was allowed before recording, and signals were digitized at 1000 Hz. Respiratory frequency (F), tidal volume (Vt), minute ventilation (Mv), and peak inspiratory flow (PIF) were calculated using ResMass software (version 1.4.2.8), with PIF serving as a key parameter for assessing inspiratory flow limitation. Respiratory signals were analyzed based on sleep state: all signals during REM sleep and randomly selected 20 s segments every 30 min during NREM sleep were analyzed. Apneas were manually scored as previously described,^[^ ^42^ ^]^ defined as a ≥90% reduction in airflow lasting at least two respiratory cycles or ≥0.7 s, with or without arousal. The apnea index (AI) was calculated as the total number of apneas divided by total sleep time (hours).

### Statistical Analysis

All data are presented as mean ± SD. Statistical analysis and visualization were performed using GraphPad Prism 8.0 and ImageJ. For normally distributed independent samples, an F‐test was used to assess homogeneity of variance, followed by an unpaired t‐test (equal variance) or Welch's corrected unpaired t‐test (unequal variance). Non‐normally distributed independent samples were analyzed using the Mann‐Whitney test. Multiple group comparisons were performed using one‐way ANOVA or two‐way ANOVA. *P < 0.05, **P < 0.01, and ***P < 0.001 were considered statistically significant, with *P* < 0.05 as the threshold for significance.

## Conflict of Interest

The authors declare no conflict of interest.

## Author Contributions

Y.W. and Q.Z. contributed equally to this work. K.H.: conceptualization, funding acquisition, writing—original draft, writing—review & editing, supervision, project administration, and resources. G.D.: conceptualization, writing—original draft, writing—review & editing, supervision, project administration, and resources. Y.W.: investigation, methodology, formal analysis, and writing— original draft, writing—review & editing. Q.Z.: investigation, methodology, formal analysis, and writing—original draft, writing—review & editing. Q.Z.: validation, software, formal analysis. X.W.: validation, software, and formal analysis. X.L.: formal analysis, validation, software, and resources. S.W.: formal analysis, validation, software, and resources. B.Z.: validation, visualization, and writing—review & editing, project administration, and resources. S.Z.: validation, visualization, and resources. D.Z.: supervision, writing—review & editing, project administration. H.L.: supervision and resources.

## Supporting information



Supporting Information

## Data Availability

The data used and/or analyzed during the current study are available from the corresponding author upon reasonable request.
