# Supplementary material for: Targeted Delivery of CNS‐Specific Hesperidin as a Leptin Sensitizer for Treating Obesity‐Associated Sleep‐Disordered Breathing
Source: Adv Sci (Weinh). 2025 Sep 9;12(45):e06182. doi: 10.1002/advs.202506182 (PMC12677648; doi:10.1002/advs.202506182)
Supplement: Supplementary file 1 — Supporting Information [file ADVS-12-e06182-s001.docx]

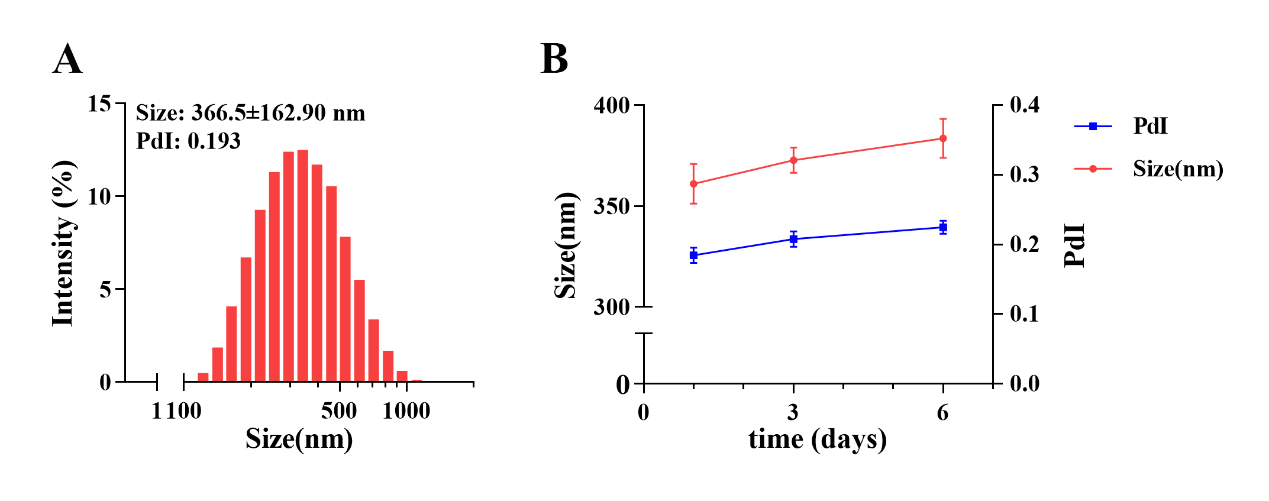
**Figure S1.** **Particle size and stability analysis of HE NPs.** (A) Initial hydrodynamic diameter and polydispersity index (PdI) of HE NPs determined by dynamic light scattering (DLS). (B) Temporal profiles of hydrodynamic diameter and PdI in PBS over 1, 3, and 6 days. Data are presented as mean ± standard deviation (SD). n = 3 per group.


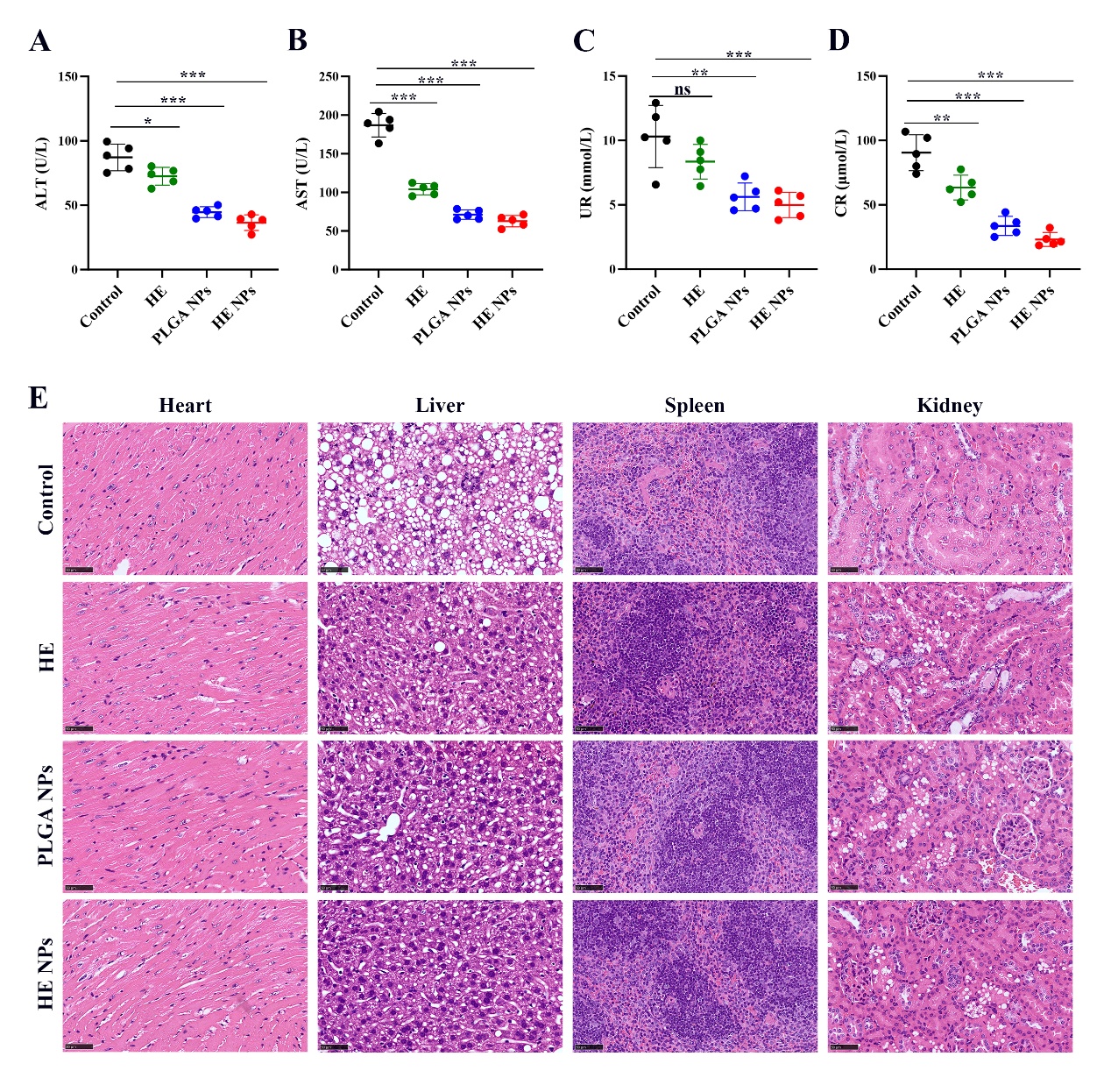


**Figure S2. Toxicity assessment in DIO mice.** (A) Alanine aminotransferase (ALT), (B) aspartate aminotransferase (AST), (C) urea (UR), and (D) creatinine (CR) levels in control, HE, PLGA NPs, and HE NPs groups after 14 days of treatment (n = 5 per group). (E) Representative H&E staining of the heart, liver, spleen, and kidneys from each group after 14 days of treatment (scale bar = 50 μm). Data are presented as mean ± standard deviation (SD). ns, not significant; *P < 0.05; ***P < 0.001. A P-value < 0.05 was considered statistically significant.


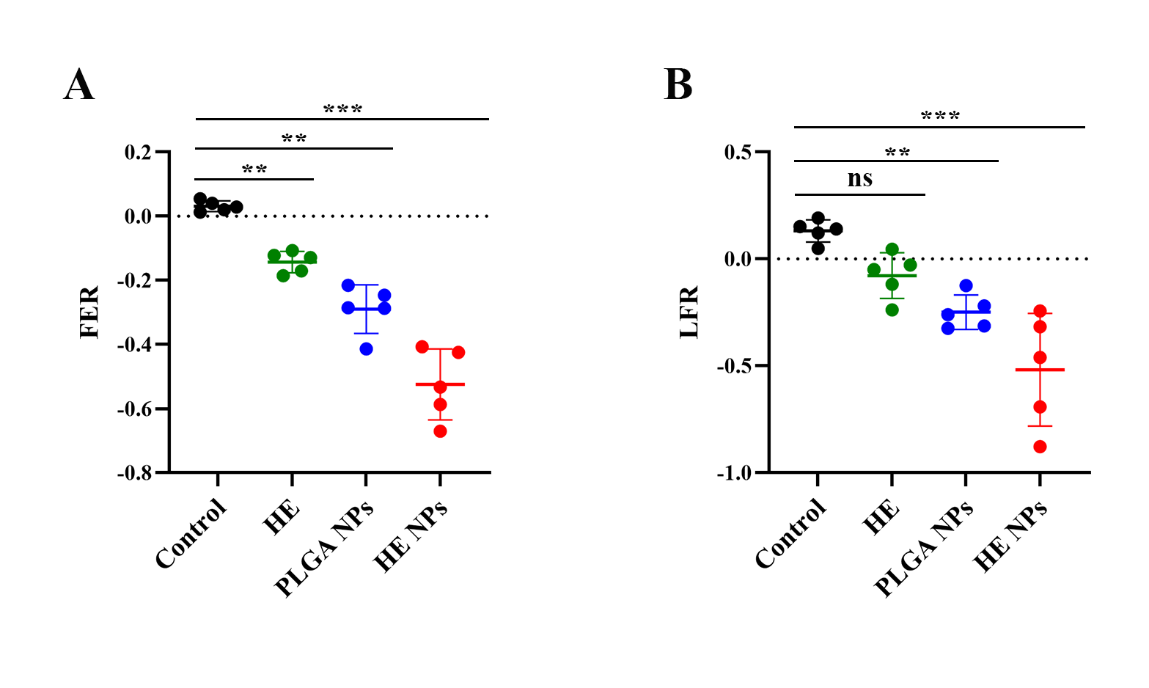
 **Figure S3. Food efficiency ratio (FER) and leptin-to-food ratio (LFR) in DIO mice.** (A) Food efficiency ratio (FER), calculated as body weight change (g) divided by cumulative food intake (g), in control, HE, PLGA NPs, and HE NPs groups (n = 5 per group). (B) Leptin-to-food ratio (LFR), calculated as the change in plasma leptin level (post-treatment value minus the average baseline value of five batch-matched mice) divided by cumulative food intake (g), in control, HE, PLGA NPs, and HE NPs groups (n = 5 per group). Data are presented as mean ± standard deviation (SD). ns, not significant; *P < 0.05; ***P < 0.001. A P-value < 0.05 was considered statistically significant.
